# Multiple myeloma with lung plasmacytoma

**DOI:** 10.4103/0970-2113.80331

**Published:** 2011

**Authors:** Rajendra Prasad, Sanjay Kumar Verma, Rakhi Sodhi

**Affiliations:** *Department of Pulmonary Medicine, Chatrapati Sahuji Maharaj Medical University (Earlier KGMU), Kanpur, India*; 1 *Department of Tuberculosis and Respiratory Diseases, G.S.V.M. Medical College, Kanpur, India*

**Keywords:** Extramedullary dissemination, lung, malignant myeloma

## Abstract

Malignant myeloma (MM) is a clonal proliferation of plasma cells with multiple osteolytic lesions. Extramedullary dissemination of multiple myeloma in lung is relatively uncommon. Hereby, we present a case of multiple myeloma with lung plasmacytoma of lung in a 45-years-old, non-smoker, female.

## INTRODUCTION

Multiple myeloma (MM) is a systemic disease process primarily involving the bone marrow. In MM, the normal bone marrow is replaced by malignant plasmacytes, which produce monoclonal proteins and this disease process mainly involves the axial skeleton. MM constitutes about 1% of all malignancies and 10% of hematologic malignancies.[1] Extra-medullary plasmacytomas form a small percentage of plasma cell tumors, and majority of which primarily occur in the head and neck. However, the occurrence of extramedullary disease is very uncommon in MM.[2] Hereby, we present a case of multiple myeloma with lung plasmacytoma of lung, in a 45-years-old, non-smoker, female.

## CASE REPORT

A 45-year-old female, non smoker was admitted to our department with complaints of right-sided chest pain and breathlessness for one year duration; loss of appetite for six-month duration; and about 6-kg weight loss over four weeks. The pain was moderate in intensity, constant, and localized primarily to the upper part of the chest wall both anteriorly and posteriorly. The pain increased to some extent on movement. She had taken antitubercular drugs for last six months.

Physical examination revealed pallor. Local examination revealed about 6 × 6 cm hard swelling, just below right breast, tender and fixed to underlying structure.

**Figure 1:**
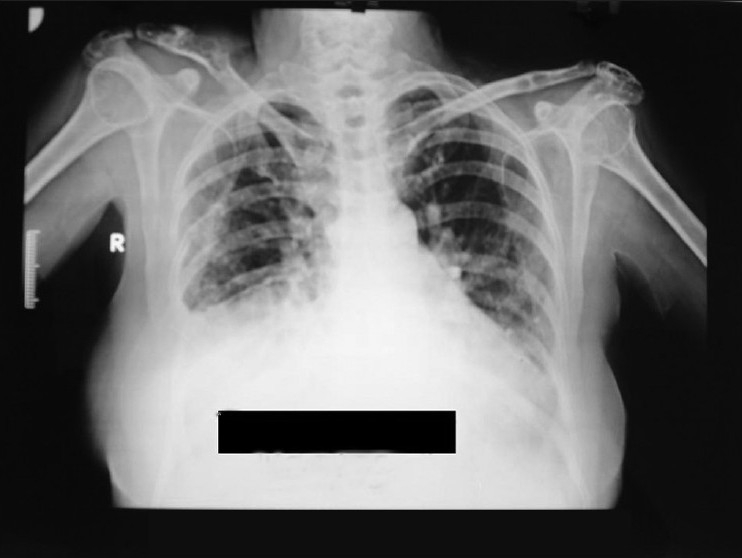
Chest radiograph revealing opacity in the right lung with lytic lesions over right clavicle and erosion of the right fifth rib.

Her chest radiograph revealed opacity in the right lung with lytic lesions over right clavicle and erosion of the right fifth rib [Figure 1].

Routine investigation showed hemoglobin: 5.1 g%, total leukocyte count: 5,500/mm^3^, differential count: neutrophils 64%, lymphocyte 34%, eosinophil 2% and platlets count: 1.7 lacs|mm^3^, serum creatinine 1.9 mg/dl and 24 h urinary protein 330 mg/dl. Her whole skeletal survey revealed no abnormality except thorax.

**Figure 2:**
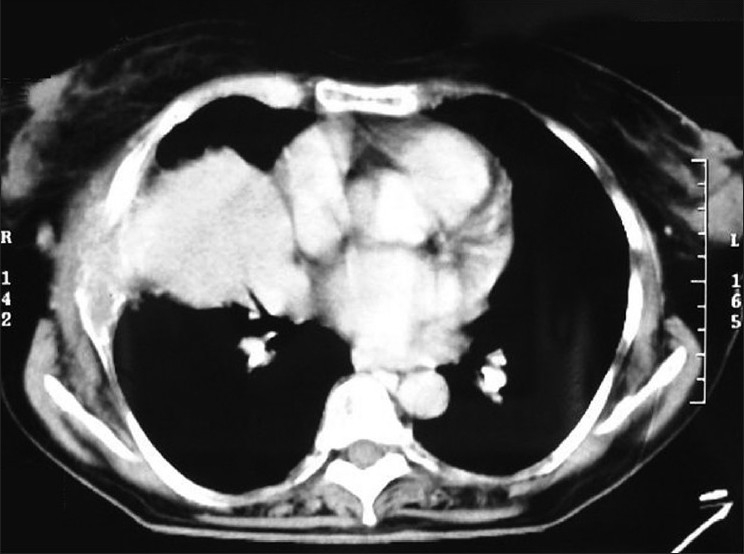
Computed tomographic scan of the thorax (CT thorax) revealing soft tissue mass in the right anterior chest wall with rib destruction and lytic lesions in lateral end of right clavicle and posterior end of fifth rib suggestive of metastaic deposits

**Figure 3:**
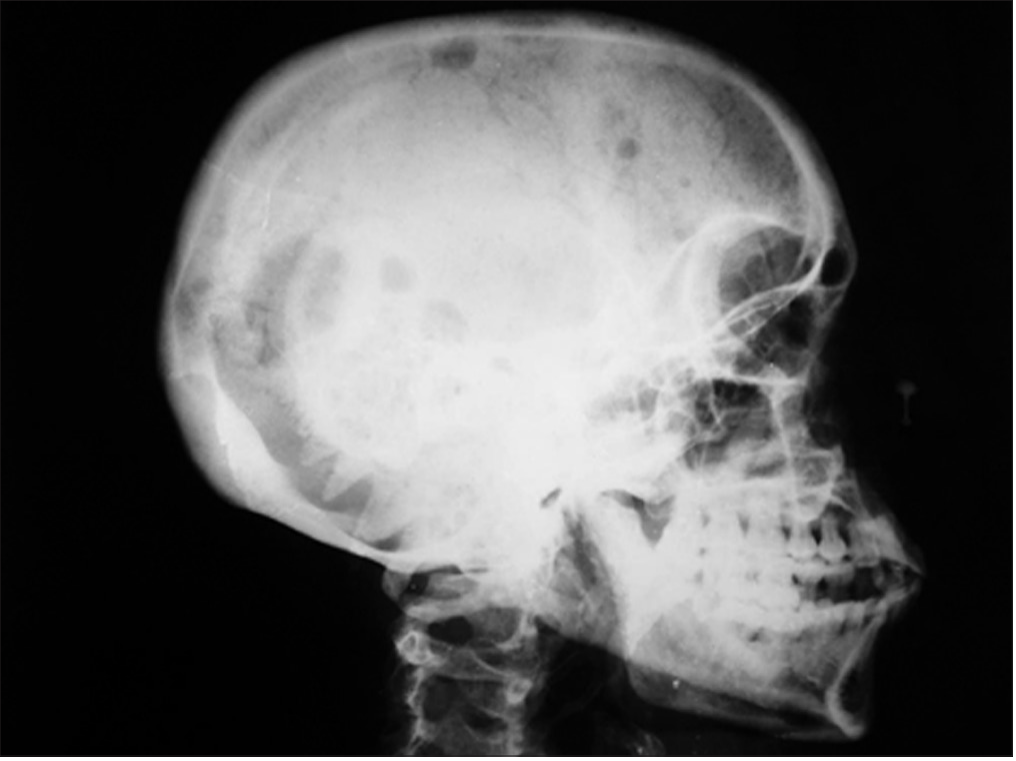
Skull radiograph revealing multiple lytic lesions

A computed tomographic scan of the thorax (CT thorax) revealed soft tissue mass in the right anterior chest wall with rib destruction and lytic lesions in lateral end of right clavicle and posterior end of fifth rib suggestive of metastatic deposits [Figure 2]. Thus, a possibility of metastatic disease was raised. Biopsy of the lung mass revealed atypical plasma cells arranged in sheets with pulmonary parenchymal cells suggestive of malignant myeloma. Her skull X-ray revealed multiple lytic lesions [Figure 3]. Serum protein electrophoresis was done which revealed raised total proteins (11 g/dl) with normal albumin, alfa-1, alfa-2, and beta globulin but markedly raised gamma globulin (5 g/dl) and the electrophoresis showed an M-spike in the beta-gamma inter zone. Serum immunoglobulin determination revealed markedly raised IgG immunoglobulin (3000 g/dl). Beta-2 microglobulin was 3815 ng/ml. Urinary examination for Bence Jones’ proteins was positive. Bone marrow biopsy revealed a hypocellular marrow with more than 75% plasma cells and reduced myeloid and erytheroid cells.

Thus, diagnosis of MM with lung plasmacytoma was made on the basis of plasma cell infiltration of the bone marrow, the lytic bone lesions, the presence of monoclonal immunoglobulins in the serum and the myeloma plasma cells in the lung mass. The patient was planned to be referred to the oncology department for chemotherapy but was not willing to undergo any form of definitive treatment.

## DISCUSSION

In multiple myeloma, bone marrow is infiltrated with aggregates of abnormal plasma cells and that leading to multifocal destructive bone lesions.

Extramedullary plasmacytoma accounts for about 3% of plasma cell malignancies and approximately 80% of which, in the upper respiratory tract namely oronasopharynx and paranasal sinuses.[2,3] But association of multiple myeloma with lung plasmacytoma is found to be extremely rare.[4,5]

The most typical thoracic manifestations of multiple myeloma are bony involvement of the thoracic cage. While other manifestations are pneumonia, intra-parenchymal mass lesions, mediastinal lymphadenopathy, reticulonodular shadows, interstitial pattern and intrapulmonary calcification (details of manifestations are given in Table 1, as reported by various authors).

**Table 1:** Comparative analysis of pulmonary manifestations in multiple myeloma by various authors

	Shin *et al*.[6]	Weber *et al*.[7]	Koss *et al*.[8]	Duggal *et al*.[5]	Oymak *et al*.[9]	Damoj *et al*.[10]	Sullivan *et al*.[4]	Present case
Type	Case report	Case report	Case report	Case report	Prospective study	Prospective study	Case report	Case report
No. of cases	2	1	5	1	38 * only 13 had lung manifestations	432 * only 11 had lung manifestations	1	1
Age/sex	*71/M *68/F	52/F	*4 males and 1 female *ages 50-79 years	60/M	*25 males and 13 female *ages 40-80 years	*8 males and 3 female *ages 39-79 years	51/M	45/F
Chest radiology	*Mass *Multiple nodule	Pulmonary calcification	*hilar mass 2 *intraparenchymal mass 3	Homogenous Opacity in UZ+MZ of right lung and erosion of right 6th rib	*Pneumonia 6 *Interstial shadows 3 *Mass lesions 2 *Multiple nodules 2	*Pleural involvement 8 *Lung parenchyma 3	Bilateral hilar lymph node + lung nodule	Mass lesion

Despite advances in the diagnosis of MM, it remains an incurable disease, because the disease follows a relapsing course in majority of patients, regardless of the treatment regimen or initial response to treatment.

Newly diagnosed patients with good performance status are best treated with autologus stem cell transplantation. These patients are treated with high dose chemotherapy (HDCT) with vincristine, melphalan, cyclophosphamide and prednisone (VMCP) alternating with vincristine, carmustine, doxorubicin and prednisone (BVAP) combined with bone marrow transplantation, it improves the response rate, even free survival and overall survival in multiple myeloma. Induction therapy in patients ineligible for transplantation (old age, coexisting conditions, poor physical condition) includes thalidomide in combination with melphalan and prednisone or melphalan and prednisolone. Recently, the management of patients with MM has been transformed by introduction of three novel agents: thalidomide, lenalidomide, and bortezomib.[11,12]

The differential diagnoses of multiple myeloma are metastatic carcinoma, lymphoma, bone neoplasm and chronic lymphocytic leukemia.[13]

The prognosis of patients with pulmonary multiple myeloma is poor.[8] This contrasts with the reports of long survival rates with primary pulmonary plasmacytomas of the lung.[9]
